# Microbiological, Nutritional, and Sensory Quality of Bread Produced from Wheat and Potato Flour Blends

**DOI:** 10.1155/2014/671701

**Published:** 2014-08-11

**Authors:** Udeme Joshua Josiah Ijah, Helen Shnada Auta, Mercy Oluwayemisi Aduloju, Sesan Abiodun Aransiola

**Affiliations:** Department of Microbiology, Federal University of Technology, P.O. Box 65, Minna 920281, Nigeria

## Abstract

Dehydrated uncooked potato (Irish and sweet) flour was blended by weight with commercial wheat flour at 0 to 10% levels of substitution to make bread. Comparative study of the microbial and nutritional qualities of the bread was undertaken. The total aerobic bacterial counts ranged from 3.0 × 10^5^ cfu/g to 1.09 × 10^6^ cfu/g while the fungal counts ranged from 8.0 × 10^1^ cfu/g to 1.20 × 10^3^ cfu/g of the sample. Coliforms were not detected in the bread. Bacteria isolated were species of *Bacillus*, *Staphylococcus*, and *Micrococcus* while fungi isolates were species of *Aspergillus*, *Penicillium, Rhizopus*, and *Mucor*. The mean sensory scores (color, aroma, taste, texture, and general acceptability) were evaluated. The color of the bread baked from WF/IPF_2_ (wheat/Irish potato flour, 95 : 5%) blend was preferred to WF (wheat flour, 100%) while WF/SPF_1_ (wheat/sweet potato flour, 100%) and WF/IPF_1_ (wheat/Irish potato flour, 90 : 10%) aroma were preferred to WF. However, the bread baked from WF, WF/IPF_2_ (wheat flour/Irish potato flour, 95 : 5%), and WF/SPF_2_ (wheat/sweet potato flour, 95 : 5%) was more acceptable than other blends. The use of hydrated potato flour in bread making is advantageous due to increased nutritional value, higher bread yield, and reduced rate of staling.

## 1. Introduction

Bread is universally accepted as a very convenient form of food that is important to all populations. Its origin dates back to the Neolithic era and is still one of the most consumed and acceptable staple food products in all parts of the world. It is a good source of nutrients, such as macronutrients (carbohydrates, protein, and fat) and micronutrients (minerals and vitamins) that are essential for human health [1].

In Nigeria, bread has become the second most widely consumed nonindigenous food product after rice. It is consumed extensively in most homes, restaurants, and hotels. It has been hitherto produced from wheat as a major raw material [1]. In Nigeria, wheat production is limited and wheat flour is imported to meet local flour needs for bakery products. Thus, huge amount of foreign exchange is used every year for import of wheat. Efforts have been made to promote the use of composite flours in which flour from locally grown crops and high protein seeds replace a portion of wheat flour for use in bread, thereby decreasing the demand for imported wheat and helping in producing protein-enriched bread [2]. Most tropical cereal grains and some tubers have been used to make composite flour for bread making [3].

Although there is now a substantial amount of composite bread, such bread still requires at least 70% wheat flour to be able to rise because wheat contains gluten [4, 5]. The successful use of composite flour has been variously reported in the literature. Olaoye [6] reported the use of composite flour of wheat, plantain, and soybeans in bread making. According to the authors, good quality and acceptable baked products could be derived from composite flours with up to certain levels of breadfruit flour substitution in wheat flour [6]. Composite flours have been used extensively in the production of baked goods. In fact, several attempts have been made to produce cookies from different types of composite flours. In countries where malnutrition poses a serious problem especially among children, composite flours which have better nutritional quality would be highly desirable [7]. It has also been reported that composite flour can be made from legumes and nuts and root and tubers such as yam, cassava, and sweet potatoes and sensory qualities of yam and sweet potatoes flours have been reported [8].

Potato is a food crop with potential for partial replacement of wheat in bread making. Uncooked potato flour prepared by low-cost solar dehydration technology has a long shelf life and high nutritional quality, which could be valuable in cereal-based human diets, including bread [9]. Sweet potato flour can serve as a source of energy and nutrients (carbohydrates, beta-carotene, and minerals) and can add natural sweetness, color, flavor, and dietary fiber to processed food products [10]. Addition of various proportions of potato flour in wheat flour can increase the nutritive values in terms of fibre and carotenoids. This also helps in lowering the gluten level and prevents coeliac disease.

The aim of this study was to replace part of the wheat flour in bread by potato flour in order to increase the fibre and other nutrients. The microbiological qualities of the bread were also assessed.

## 2. Materials and Methods

### 2.1. Collection of Samples

The dry starchy yellow-fleshed cultivar of sweet potato (*Ipomea batatas*) and Irish potato (*Solanum tuberosum*) and wheat flour were purchased from a local market in Offa, Kwara State, Nigeria, and the Central Market, Minna, Niger State, Nigeria, respectively, in polythene bags, and transported to the laboratory for processing.

### 2.2. Production of Potato Flour

The potatoes were washed under running tap water (to free them of adhering soil particles), air dried, and stored at 12°C before use. The potato flours were prepared from solar-dried slices according to the method outlined in [9, 11].

### 2.3. Baking Process

Eight blend formulations (Table 1) were baked using the straight dough method [12]. The Baking formula was 50.4 g–53.2 g wheat flour (90–95%), 1.8 g–5.6 g potato flour (5–10%), 36% water, 3.4% sugar, 1% skim milk powder, 1% salt and 1% yeast, similar to that of [13]. All ingredients were mixed in a Kenwood mixer (Model A 907D) for 5 minutes. The dough was fermented in bowls, covered with wet clean Muslin cloth for 55 minutes at warm temperature, punched, scaled to 250 g dough pieces, proofed in a proofing cabinet at 30°C for 90 minutes and 85% relative humidity, and baked at 250°C for 30 minutes [14]. The baked bread samples were then depanned, cooled at ambient temperature and put in ziploc bags prior to analysis.

### 2.4. Microbiological Analysis of Bread Produced

Total mesophilic (total viable bacterial counts) and fungi counts (yeast and mould counts) were carried out on the bread samples to determine the microbial load of the samples as described by American Public Health Association [15]. Bread samples were prepared by mashing and mixing in peptone water. Subsamples were diluted decimally and 0.1 mL aliquots were spread plated on nutrient agar (NA), MacConkey agar (MCA), and potato dextrose agar (PDA) for the enumeration of aerobic viable bacteria, coliforms, and fungi, respectively. The NA and MCA plates were incubated at 37°C for 24–48 hours while PDA plates were incubated at room temperature (28 ± 2°C) for 3–5 days. The colonies were then counted and expressed as colony forming units per gram (cfu/g) of samples. All counts were done in duplicate using the Stuart scientific colony counter. Observed colonies were subcultured repeatedly on media used for primary isolation to obtain pure cultures.

### 2.5. Characterization and Identification of Isolates

The bacterial isolates were characterized using Gram reaction and biochemical tests and were identified by comparing their characteristics with those of known taxa as outlined in Bergey's Manual of Systematic Bacteriology [16]. The fungal isolates were characterized based on macroscopic and microscopic examination and identified using the scheme of [17].

### 2.6. Quality Evaluation of Bread

#### 2.6.1. Moisture

Moisture content of the bread was determined using the procedure described by AOAC [18].

#### 2.6.2. Crude Protein

Protein was determined using the micro-Kjeldahl method AOAC [18]. The concentration of protein in the digested sample was determined spectrophotometrically and calculated as

(1) %  crude  protein=(titre  of  sample−blank)×0.01×14.007×6.2510×weight  of  sample×100.

#### 2.6.3. Crude Fat

This was carried out using the standard method of AOAC [18].

#### 2.6.4. Crude Fibre and Ash Content

Determination of the crude fibre and the ash content in the bread samples was carried out using the standard methods described by AOAC [18].

#### 2.6.5. Carbohydrate

Carbohydrate was determined using estimation by difference AOAC [18]. The crude fibre, crude protein, and the fat content were subtracted from organic matter; the remainder accounted for carbohydrates:

(2) $$\begin{array}{c} \%\;\;\text{carbohydrate}=100-\text{protein}\left(\%\right)+\text{fat}\left(\%\right)+\text{ash}\left(\%\right). \end{array}$$

#### 2.6.6. Sensory Evaluation of Bread Produced

Sensory evaluation was preformed 24 hours after baking to evaluate loaf color, crust, aroma, crumb texture, taste, and overall acceptability of the bread sample. A panel of ten judges (using a questionnaire) of regular bread consumers using the Hedonic scale product was set up. The panel was set up in three sets (to obtain three replicates) and the sensory scores were analyzed statistically.

#### 2.6.7. Storage of Bread

The bread samples were stored under ambient temperature (26°C–33°C) and observed for 10 days. Visual observations for mould growth were carried out on the samples stored.

### 2.7. Statistical Analysis

Statistical analyses were performed using the SPSS (version 20). Difference in proximate composition and sensory scores was detected using one-way analysis of variance (ANOVA). A significance level of (*P* < 0.05) was used for the study.

## 3. Results

### 3.1. Microbial Counts and Identification

The total bacterial counts of the bread samples ranged from 3.0 × 10^5^ cfu/g to 1.0 × 10^6^ cfu/g with the highest being recorded for bread made from 100% sweet potato flour (SPF) while the lowest counts (3.0 × 10^5^ cfu/g) were obtained in bread made from 100% Irish potato flour (IPF). Bacteria were not detected in bread baked from 100% wheat flour (WF); see WF/SPF_1_, WF/SPF_2_, and WF/IPF_2_ in Table 2. The fungi counts ranged from 8.0 × 10^1^ cfu/g to 1.2 × 10^3^ cfu/g with the highest counts recorded for bread baked from WF/SPF_2_ while the lowest counts (8.0 × 10^1^ cfu/g) were observed in bread baked from SPF/IPF (Table 2). Fungi were not detected in bread baked from WF, WF/SPF_1_, WF/SPF_2_, and WF/IPF_2_. Coliforms were not detected in any of the bread samples analyzed. Three species of bacteria were isolated which include* Bacillus subtilis*,* Micrococcus* sp., and* Staphylococcus aureus*. For the fungi,* Aspergillus niger*,* Penicillium stolonifer*,* Rhizopus nigricans*, and* Mucor* sp. were isolated.

### 3.2. Proximate Composition of Bread

The highest moisture content of 16.0% was obtained in WF/IPF_1_ bread while the lowest moisture level (11.50%) was obtained in SPF/IPF. The respective values obtained in WF, SPF, IPF, and WF/IPF bread were 15.15%, 15.31%, 14.945, and 12.63% (Table 3). The highest value for lipid (4.20%) was obtained in WF/SPF_1_ bread while the lowest value of 2.35% was recorded in SPF/IPF bread. The crude fibres of 1.33%, 1.84%, 1.42%, 0.90%, 2.00%, 1.87%, 1.92%, and 2.0% were obtained in the WF, SPF, IPF, SP/IPF, WF/SPF_1_, WF/IPF_1_, WF/SPF_2_, and WF/IPF_2_ bread samples. The highest ash content (3.75%) was obtained in IPF bread, while the lowest ash content (1.50%) was obtained in SP/IPF bread. The carbohydrate content was higher in WF/SPF_1_ (87.08%) than in the rest of the flour blends, but it was, however, the least (70.10%) in SPF/IPF bread (Table 3). The statistical analysis of the data revealed that the proximate composition of the various blends was significantly different (*P* < 0.05).

### 3.3. Sensory Evaluation of Bread

The mean sensory scores for each quality attribute evaluated (color, aroma, taste, crust, texture, and general acceptability) of the bread samples prepared from the wheat/potato blends are presented in Table 4. The statistical analysis of the data showed that there were significant differences (*P* < 0.05) among the wheat/potato blends with the exception of the WF and WF/SPF_2_ for color, as well as WF/SPF_2_ and WF/IPF_2_ for aroma. The scores also indicated that bread baked from WF was more acceptable than that from other blends. However, this was closely followed by bread baked from WF/IPF_2_ and WF/SPF_2_ blends.

### 3.4. Storage of Bread

The bread produced lasted for 6–8 days (Table 5) before obvious spoilage was noticed. WF, WF/SPF_2_, and WF/IPF_2_ lasted for 6 days while IPF and WF/IPF_1_ lasted for 7 days before spoilage occurred. It was also observed that SPF, SPF/IPF, and WF/SPF_1_ lasted for 8 days before spoilage set in (Table 5). Spoilage was indicated by black, yellow, and green coloration on the bread (suspected to be mold growth). When the black, yellow, and green coloring matters were stained and examined under the light microscope, they consisted of* Aspergillus flavus*,* Penicillium* sp.,* Rhizopus stolonifer*, and* Mucor mucedo*.

## 4. Discussion

Bacteria and fungi were not detected in some of the bread samples produced. These are within the limit set by the Standard Organization of Nigeria, which states that the counts of aerobic bacteria must not exceed 100 cfu/g and coliform growth must not be detected in bread samples. This shows that such bread is safe for consumption as there is no fecal contamination. The high bacteria population in SPF (100% sweet potato flour) could be due to the abundance of moisture and nutrient in the potato bread, which provide for a favorable condition for growth. The bacteria isolated from the bread samples included species of* Bacillus*,* Micrococcus*, and* Staphylococcus*. Bacteria have the potential to contaminate baked products. The presence of the different bacterial species in the samples could have evolved during baking or from the raw ingredients used, for example, flour, sugar, and yeast.* Bacillus* sp. form spores which enable the bacteria to survive unfavorable conditions such as heating [19]. K. Talaro and A. Talaro [20] reported that* Staphylococcus* species are widely distributed in the environment and occur on the skin and nostrils of humans, from where the organisms can contaminate food.

The fungi counts were higher in bread made from SPF/IPF (50 : 50%) than all other samples probably because of raw materials, processing, handling, and storage. The fungi isolated include species of* Aspergillus*,* Penicillium*,* Rhizopus*, and* Mucor*. These organisms could have been introduced at the different stages of bread production. This finding is in line with the study of Daniyan and Nwokwu [21] who identified similar organisms in bread. These organisms could be responsible for the spoilage of bread.

The moisture content of food goes a long way in suggesting the shelf life of the product. The moisture content of bread made from wheat/Irish potato flour (WF/IPF, 90 : 10%) was higher than that of the other flour blends. This may be due to the processing methods the samples were exposed to. The values of the other blended samples fall within the acceptable moisture limit for dry products (15%). Adeleke and Odedeji [11] obtained similar results. Moisture is a very important factor in the keeping quality of bread and high moisture can have an adverse effect on storage stability [22]. The bread sample having the highest moisture content may therefore have reduced shelf life in comparison with other samples.

Increase in the level of potato flour resulted in decrease in the protein content from 12.25% in 100% wheat flour bread (WF) to 3.75% in bread made from 50 : 50% sweet potato/Irish potato flour (SPF/IPF). This may have been due to the low protein content of the potato flour which must have diluted the protein content of the wheat flour, thus reducing the protein level of the mixed flour. Combination of WF and IPF (95 : 5%) resulted in an increase in the protein content of the bread from 4.3% to 14.00%. This is similar to the earlier findings where protein content of snacks reduced with supplementation with starch based products [6] for bread fruit flour, [23] for plantain, and [24] for soy flour. The sudden increase in the protein content with supplementation with 5% Irish PF shows that PF can be incorporated into bread at high supplementation level and still retain its nutrient content similar to 100% WF bread.

Blending of wheat flour with 10% potato flour resulted in an increase in the fat content. This could be due to the fact that Irish potato contains about 1–4% fat which could have been responsible for the slight increase in the fat content. The value of ash in bread made from wheat flour blended with potato flour was low compared to the ash content in bread made from wheat flour. The value of crude fibre ranged from 0.90% to 2.01%. The crude fibre was above the 1.5% maximum allowable fibre content of bread flour as stated by Omole [25] but fell within the 2.0% recommended by Nigerian Raw Materials Research and Development Council [2]. Similar results were observed by Raji [26] who also recorded low ash and crude fibre contents in cookies made by using potato flour. High ash and crude fibre contents in food depict that the material is difficult to digest in the human body.

The carbohydrate content of bread increased with addition of potato flour to the wheat flour. This may be due to higher carbohydrate content in potato than in wheat. Cereals store starch as a source of energy and are low in protein, fat, and ash. Madukwe et al. [22] recorded similar results. The high level of carbohydrate is desirable in baked products because on heating starch granules in the presence of water, it swells and forms a gel which is important for the characteristic texture and structures of baked goods [8].

The mean sensory scores of quality attributes of the products indicated that, generally, panelists expressed preference for three bread samples out of the eight presented. The bread samples were those made from WF (100% wheat flour), WF/SPF_2_ (wheat/sweet potato flour 95 : 5%), and WF/IPF_2_ (wheat/Irish potato flour, 95 : 5%). Bread made from SPF/IPF (50 : 50% sweet potato/Irish potato flour), IPF (100% Irish potato flour), and SPF (100% sweet potato flour) was highly rejected by the panelists. This shows that using either sweet or Irish potato flour alone to bake bread will not be accepted, except if it is blended with wheat flour. Bread made from potatoes alone has unpleasant color and is not usually soft.

The results of the storability of bread showed that bread made from 100% wheat flour, 10% sweet potato blended bread, and bread made from 50 : 50% Irish/sweet potato could not stay longer than six days due to the presence of fungi, while other samples lasted 7-8 days. This shows that the use of 5% dehydrated potato flour in bread making reduces the rate of staling as a result of the ability to retain moisture in them.

## 5. Conclusion

The incorporation of potato flour to wheat flour improved the nutritional value of the bread. Bacteria and molds common in the environment contaminated the bread and led to its spoilage after 6–8 days; however, sensory evaluation indicated that consumer acceptable bread could be substituted with wheat flour and dehydrated uncooked potato flour in commercial bread making without sacrificing consumer acceptability. With the use of potato flour in bread making, the cost of bread production could be less.

## Figures and Tables

**Table 1:** Wheat and potato flour blends for bread making.

Sample	Wheat/potato flour blends
A	Wheat flour, WF (100%)
B	Sweet potato flour, SPF (100%)
C	Irish potato flour, IPF (100%)
D	Sweet potato/Irish potato flour, SPF/IPF (50 : 50%)
E	Wheat/sweet potato flour, WF/SPF_1_ (90 : 10%)
F	Wheat/Irish potato flour, WF/IPF_1_ (90 : 10%)
G	Wheat/sweet potato flour, WF/SPF_2_ (95 : 5%)
H	Wheat/Irish potato flour, WF/IPF_2_ (95 : 5%)

**Table 2:** Microbial counts in freshly baked bread.

Sample	Bacteria (cfu/g)	Fungi (cfu/g)
A	NG	NG
B	1.0 × 10^6^	1.2 × 10^2^
C	3.0 × 10^5^	5.0 × 10^2^
D	4.8 × 10^5^	1.2 × 10^3^
E	NG	NG
F	6.8 × 10^5^	8.0 × 10^1^
G	NG	NG
H	NG	NG

Cfu/g: colony forming units per gram; NG: no growth detected; A: wheat flour, WF (100%); B: sweet potato flour, SPF (100%); C: Irish potato flour, IPF (100%); D: sweet potato/Irish potato flour, SPF/IPF (50 : 50%); E: wheat/sweet potato flour, WF/SPF_1_ (90 : 10%); F: wheat/Irish potato flour, WF/IPF_1_ (90 : 10%); G: wheat/sweet potato flour, WF/SPF_2 _(95 : 5%); H: wheat/Irish potato flour, WF/IPF_2 _(95 : 5%).

**Table 3:** Proximate composition of freshly baked bread.

Sample	Moisture content (%)	Crude protein (%)	Crude fibre (%)	Ash (%)	Lipid (%)	Carbohydrates (%)
A	15.15 ± 0.01^c^	12.25 ± 0.01^b^	1.33 ± 0.01^g^	3.41 ± 0.58^c^	2.67 ± 0.58^g^	80.34 ± 0.01^f^
B	15.31 ± 0.01^b^	8.10 ± 0.01^d^	1.84 ± 0.01^e^	3.60 ± 0.58^b^	3.01 ± 0.58^e^	83.45 ± 0.58^c^
C	14.94 ± 0.01^e^	7.00 ± 0.56^e^	1.42 ± 0.01^f^	3.75 ± 0.58^a^	3.10 ± 0.58^d^	84.73 ± 0.58^b^
D	11.50 ± 0.01^h^	3.75 ± 0.58^g^	0.90 ± 0.01^h^	1.50 ± 0.02^h^	2.35 ± 0.58^h^	70.10 ± 0.01^h^
E	12.63 ± 0.01^g^	4.38 ± 0.58^f^	2.00 ± 0.58^b^	2.35 ± 0.58^f^	4.20 ± 0.58^a^	87.08 ± 0.58^a^
F	16.00 ± 0.11^a^	10.50 ± 0.58^c^	1.87 ± 0.02^d^	1.81 ± 0.01^g^	3.33 ± 0.08^c^	82.49 ± 0.58^d^
G	14.21 ± 0.01^f^	10.50 ± 0.58^c^	1.92 ± 0.58^c^	2.80 ± 0.58^d^	3.47 ± 0.02^b^	81.31 ± 0.58^e^
H	15.01 ± 0.56^d^	14.00 ± 1.15^a^	2.01 ± 0.06^a^	2.73 ± 0.58^e^	2.98 ± 0.58^f^	78.28 ± 0.01^g^

Values are means ± standard error of three replicates. Different superscript in the same column indicates significant differences at *P* < 0.05.

A: wheat flour, WF (100); B: sweet potato flour, SPF (100%); C: Irish potato flour, IPF (100%); D: sweet potato/Irish potato flour, SPF/IPF (50 : 50%); E: wheat/sweet potato flour, WF/SPF_1_ (90 : 10%); F: wheat/Irish potato flour, WF/IPF_1_ (90 : 10%); G: wheat/sweet potato flour, WF/SPF_2_ (95 : 5%); H: wheat/Irish potato flour, WF/IPF_2_ (95 : 5%).

**Table 4:** Mean sensory scores from taste panel of bread baked from different flour blends.

Quality attribute	Bread samples							
	A	B	C	D	E	F	G	H
Color	8.5 ± 0.12^b^	3.4 ± 0.12^e^	2.7 ± 0.17^g^	3.0 ± 0.12^f^	7.9 ± 0.06^d^	8.0 ± 0.12^c^	8.5 ± 0.06^b^	9.0 ± 0.17^a^
Taste	8.7 ± 0.12^a^	3.6 ± 0.12^e^	2.8 ± 0.12^g^	3.2 ± 0.12^f^	8.0 ± 0.12^d^	7.8 ± 0.12^c^	8.5 ± 0.12^b^	8.2 ± 0.06^c^
Aroma	8.4 ± 0.17^c^	3.6 ± 0.06^e^	2.1 ± 0.06^g^	3.0 ± 0.12^f^	8.9 ± 0.06^a^	8.7 ± 0.12^b^	8.2 ± 0.06^d^	8.3 ± 0.09^d^
Texture	8.5 ± 0.08^b^	3.2 ± 0.06^f^	2.6 ± 0.07^h^	2.9 ± 0.06^g^	7.9 ± 0.06^d^	7.7 ± 0.11^e^	9.0 ± 0.06^a^	8.3 ± 0.12^c^
Crust	8.4 ± 0.23^b^	2.8 ± 0.12^d^	2.3 ± 0.06^f^	2.8 ± 0.12^e^	7.6 ± 0.06^c^	8.7 ± 0.12^a^	8.7 ± 0.06^a^	8.7 ± 0.06^a^
Acceptability	8.6 ± 0.12^a^	2.6 ± 0.06^f^	2.4 ± 0.12^g^	1.9 ± 0.06^h^	7.7 ± 0.06^e^	8.0 ± 0.12^d^	8.2 ± 0.06^c^	8.5 ± 0.06^b^

Values are means ± standard error of three replicates. Different superscript in the same row indicates significant differences at *P* < 0.05.

A: wheat flour, WF (100%); B: sweet potato flour, SPF (100%); C: Irish potato flour, IPF (100%); D: sweet potato/Irish potato flour, SPF/IPF (50 : 50%); E: wheat/sweet potato flour, WF/SPF_1_ (90 : 10%); F: wheat/Irish potato flour, WF/IPF_1_ (90 : 10%); G: wheat/sweet potato flour, WF/SPF_2_ (95 : 5%); H: wheat/Irish potato flour, WF/IPF_2_ (95 : 5%).

**Table 5:** Length of time bread produced remained whole.

Bread made from	Spoilage of bread started after (days)
Wheat flour, WF (100%)	6
Sweet potato flour, SPF (100%)	8
Irish potato flour, IPF (100%)	7
Sweet potato/Irish potato flour, SPF/IPF (50 : 50%)	6
Wheat/sweet potato flour, WF/SPF_1_ (90 : 10%)	6
Wheat/Irish potato flour, WF/IPF_1_ (90 : 10%)	8
Wheat/sweet potato flour, WF/SPF_2_ (95 : 5%)	8
Wheat/Irish potato flour, WF/IPF_2_ (95 : 5%)	7
